# Appendiceal Mucinous Neoplasm in a De Garengeot Hernia: A Case Report

**DOI:** 10.7759/cureus.75356

**Published:** 2024-12-09

**Authors:** Mhd Anas Murad, Manoj Jacob, Rebecca Himpson

**Affiliations:** 1 General Surgery, Southend University Hospital, Mid and South Essex National Health Service (NHS) Foundation Trust, Southend-on-Sea, GBR

**Keywords:** de garengeot's hernia, femoral hernia, groin lump, lamn, low-grade appendiceal mucinous neoplasm

## Abstract

De Garengeot's hernia, a femoral hernia containing an incarcerated appendix, is rarely encountered in clinical practice. We report a case of a 62-year-old woman presenting with a right groin lump. A computed tomography scan reported a right inguinal hernia containing fluid with no bowel involvement. Exploration of the right groin revealed a femoral hernia containing an inflamed appendix. Appendicectomy and suture repair of the femoral canal were performed, with histology showing a low-grade appendiceal mucinous neoplasm, which is also rare. After a discussion during the colorectal multidisciplinary team meeting and taking into consideration the patient’s wishes, a decision was made regarding a complete right hemicolectomy. Histology from the subsequent surgery did not demonstrate any adverse features, and the patient was enrolled in the surveillance program. This case highlights the rarity of this finding and underscores the importance of tailoring surgical approaches based on the intraoperative findings aided by preoperative imaging and a thorough understanding of the anatomy.

## Introduction

Femoral hernias represent only 4% of all groin hernias. They are significant due to the greater risk of incarceration and strangulation. The appendix is an unusual finding in 0.8% of all femoral hernias [1,2].

The first description of an appendix within an incarcerated femoral hernia was made by the French surgeon René Jacques Croissant De Garengeot in 1731, now known as De Garengeot's hernia [3]. ​This rare condition represents less than 1% of surgically treated hernias, with acutely inflamed or perforated appendicitis occurring in only 0.08%-0.13% [4,5].

Appendiceal tumors are also uncommon, identified in less than 1% of appendicectomy specimens [6].​ These tumors include epithelial, neuroendocrine, mesenchymal, and lymphomas. One of the most common tumors of the appendix is low-grade appendiceal mucinous neoplasms (LAMNs). Mucinous neoplasms of the appendix are a diverse group, ranging from benign tumors to malignant mucinous adenocarcinomas [7]. The Identification of such pathology within a De Garengeot's hernia is exceptionally rare and presents unique challenges in the diagnostic and management process [5]. Here, we report a case of a De Garengeot's hernia containing an incidental finding of an LAMN.

## Case presentation

A 62-year-old woman presented with a painful right groin lump for five weeks. She did not have any specific gastrointestinal-related symptoms. Medical history otherwise included asthma and hypertension. Clinical examination was suggestive of an incarcerated femoral hernia. Blood tests were unremarkable (Table 1).

**Table 1:** Blood tests on initial presentation HCT: hematocrit; RCC: red cell count; RDW: red cell distribution width; MCV: mean corpuscular volume; MCH: mean corpuscular hemoglobin

Parameter	Value	Measuring unit	Reference range
Full blood count			
Hemoglobin	133	g/L	115-165
White blood cell count	8.9	10^9^/L	4.0-11.0
Platelet count	476	10^9^/L	150-400
HCT	0.39	L/L	0.37-0.46
RCC	4.36	10^12^/L	3.8-5.8
RDW	12.8	%	11.0-14.8
MCV	88.7	fL	80-100
MCH	30.4	pg	27.0-32.0
Neutrophil count	6.41	10^9^/L	1.7-7.5
Lymphocyte count	1.63	10^9^/L	1.0-4.5
Monocyte count	0.70	10^9^/L	0.2-0.8
Eosinophils	0.09	10^9^/L	0.0-0.4
Basophils	0.07	10^9^/L	0.0-0.1
Urea and electrolytes			
Sodium	138	mmol/L	133-146
Potassium	4.3	mmol/L	3.5-5.3
Urea	4.5	mmol/L	2.5-7.8
Creatinine	51	µmol/L	45-85

**Figure 1:**
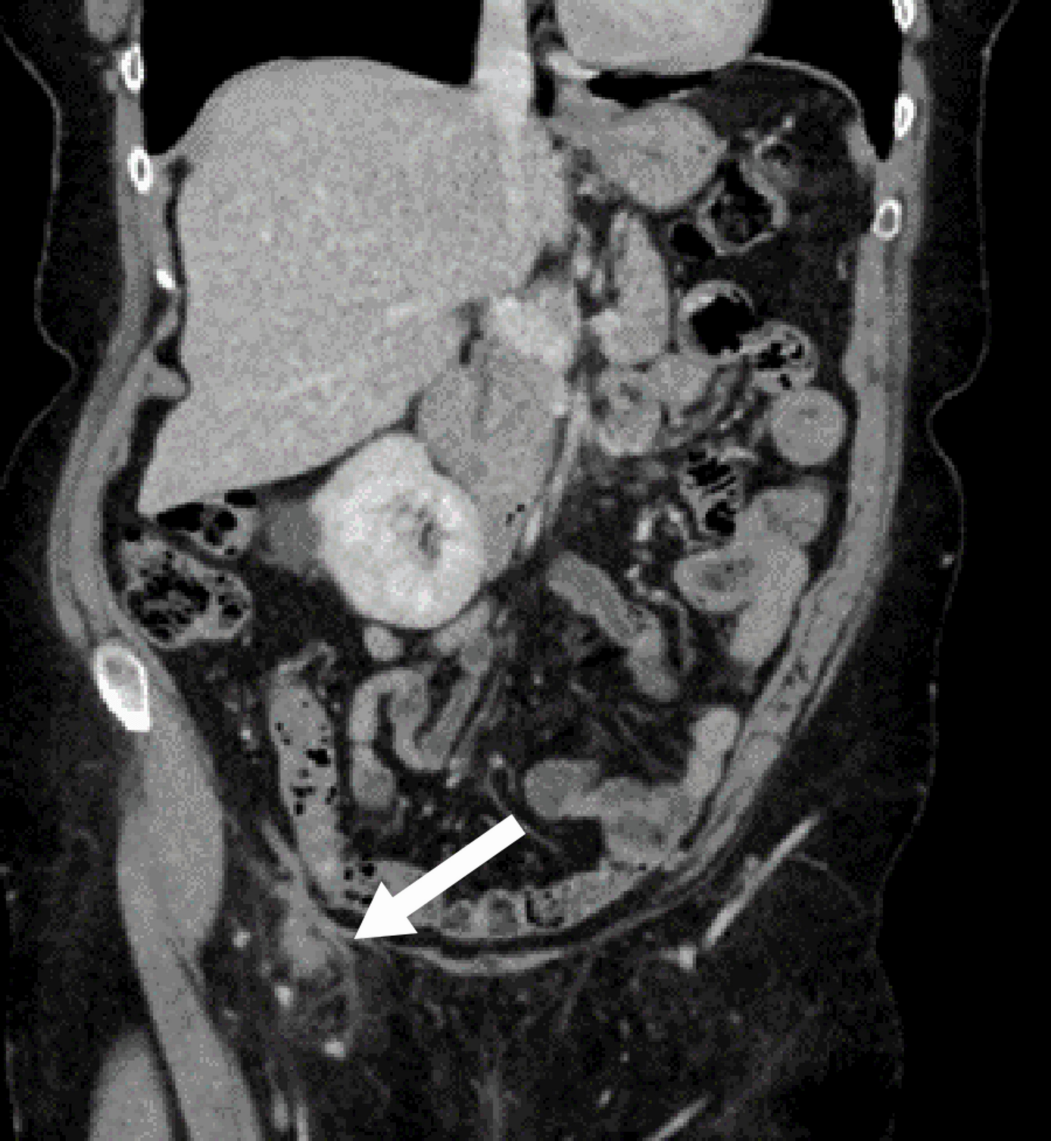
Coronal view of the CT scan of the abdomen and pelvis showing a right groin swelling (white arrow) that contains a pocket of fluid with a sac measuring 41 × 36 mm with no involvement of the bowel loops CT: computed tomography

**Figure 2:**
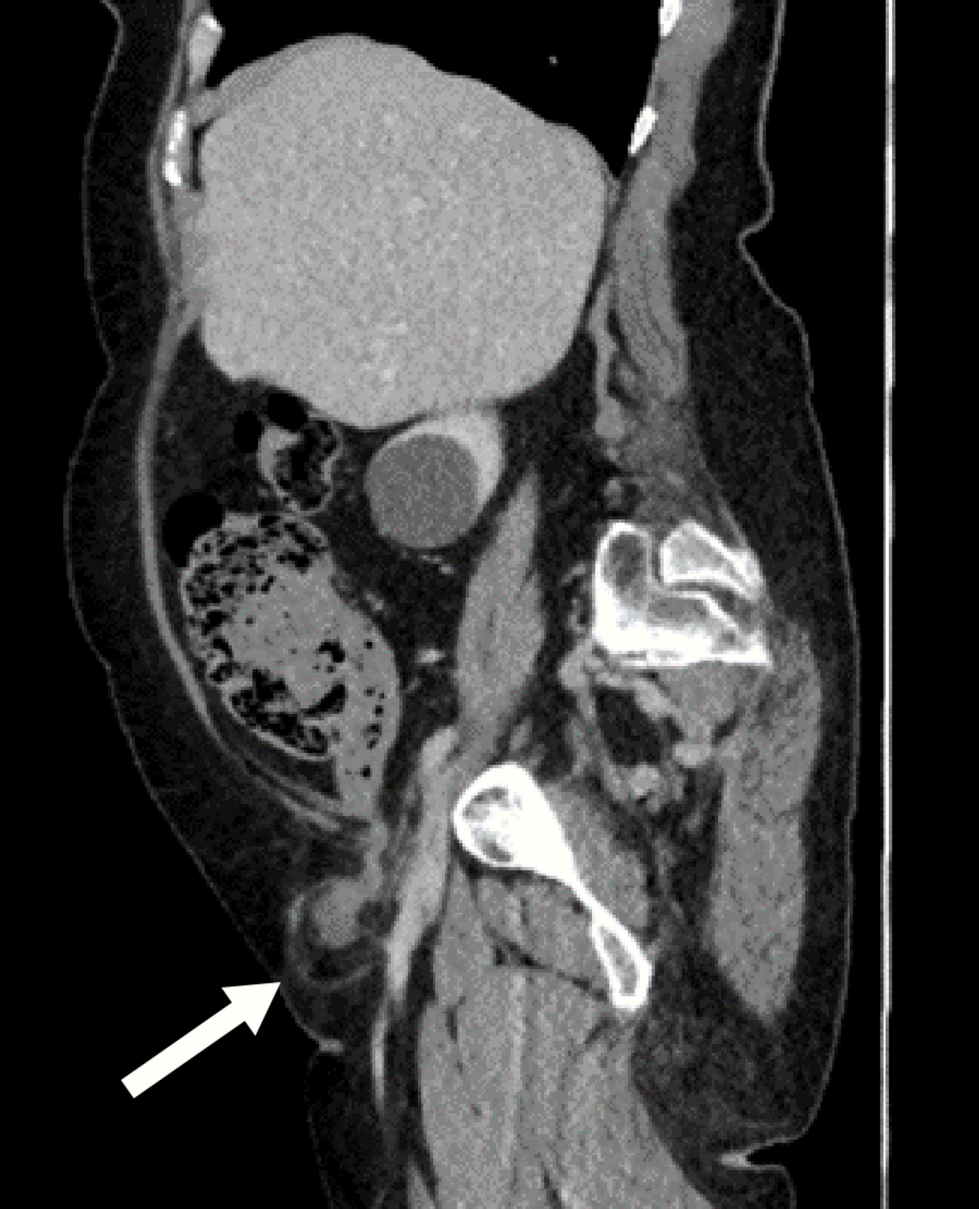
Sagittal view of the CT scan of the abdomen and pelvis showing a right groin swelling (white arrow) that contains a pocket of fluid with a sac measuring 41 × 36 mm with no involvement of the bowel loops CT: computed tomography

A computed tomography (CT) scan of her abdomen and pelvis was done to identify the nature of the lump and to delineate the anatomy to aid in the surgical planning and management. The CT scan reported a right inguinal hernia containing a pocket of fluid with a sac measuring 41 × 36 mm and no involvement of the bowel loops (Figures 1, 2).

In view of the discrepancy between the clinical findings and the imaging report, an open inguinal approach was adopted, with femoral hernia considered a differential diagnosis. Exploration of the right groin revealed a femoral hernia containing inflamed preperitoneal fat and an inflamed appendix. The peritoneal cavity was accessed through the same incision, appendicectomy was completed, and suture repair of the femoral hernia was performed.

Histological examination of the appendix showed fibrous obliteration of the lumen at the tip of the appendix. The proximal part of the appendix showed features compatible with an LAMN with no invasion, Grade 1, but involving the proximal resection margin. Further colonoscopy was arranged and was normal.

Discussion in the colorectal multidisciplinary team meeting (MDT), in view of the pathology report and the unremarkable colonoscopy, advised that the patient could undergo either a complete right hemicolectomy or involvement in a surveillance program. Taking into consideration the patient’s wishes, a decision was made for a complete right hemicolectomy. The surgery was completed laparoscopically with no complications, and the patient recovered well. There was no residual disease in the specimen; thus, the patient was enrolled in the nurse-led surveillance program for the next 10 years for LAMN.

## Discussion

In this case, the key findings include the presence of an LAMN within a femoral hernia involving the proximal resection margin. Following the MDT's advice and the patient's wishes, a complete right hemicolectomy was carried out. De Garengeot's hernia is a rare type of femoral hernia in which the appendix is located within the hernial sac. It accounts for less than 1% of all femoral hernias [8]. Given its rarity, it is often misdiagnosed for other more common types of hernias [9,10]. Thus, delayed management and surgical intervention can lead to a poorer prognosis [11].

This type of hernia is more common in women, with painful groin swelling being the most common presenting symptom. A review of all reported cases written in English demonstrated the complexity of the diagnosis, showing that it was made preoperatively in only 34.7% of cases [1].​

There is no standardized procedure for femoral hernia repair. However, the decision of the approach is affected by several factors, including preoperative diagnosis, emergency or elective presentation, and the nature of the pathology expected. In cases where the diagnosis is known preoperatively, an approach that allows entry to the peritoneal cavity is encouraged, varying between a high inguinal approach and laparoscopic approaches. In some instances, hybrid approaches are also adopted [1,5].

In the context of our case, a discrepancy between the clinical and radiological diagnosis was encountered. This highlights the importance of maintaining a broad differential diagnosis in the diagnostic and management processes.

When the appendix is inflamed, most surgeons prefer a primary repair with nonabsorbable sutures and no mesh. In the cases included in a literature review, mesh was limited to a few instances, mostly when a macroscopically normal appendix was found [1].

This report presents an extremely rare scenario in which an appendiceal tumor is found within a De Garengeot's hernia. To our knowledge, only one other case with similar findings was reported in the literature. In that study, the histological analysis revealed an LAMN of the distal appendix [5].

Due to the preoperative diagnostic difficulties and the wide variability in the surgical management of such a hernia, researchers have recommended the need for a tailored surgical approach based on the intraoperative findings aided by preoperative imaging. In addition, they have also emphasized the importance of the histological examination in guiding the ongoing management of appendiceal tumors including the critical role of the MDT [1,11].

## Conclusions

This case report discusses a rare diagnosis of a De Garengeot's hernia with an LAMN within the appendix. While preoperative imaging can aid in the diagnostic process, this case highlights its limitations in accurately diagnosing such a rare presentation. Thus, careful consideration and interpretation of imaging modalities are useful in cases where the diagnosis is unclear.

Tailored surgical approaches are based on a good understanding of the anatomy of hernias and the knowledge that imaging reports are not always correct. The involvement of the MDT in managing cases complicated by appendiceal neoplasm is mandatory. This allowed careful communication with the patient so that they can make an informed decision regarding further treatment.

Adding this rare presentation to the literature will allow researchers to explore this rare occurrence further and set a cornerstone for developing standardized guidelines for the management of similar cases, ultimately optimizing surgical approaches and improving patient outcomes.

This management led to a favorable outcome for this patient, including a complete recovery following completion surgery with long-term surveillance planned.
